# Supplementary material for: Traffic conflict identification method on curved road based on Frenet coordinate system
Source: PLoS One. 2026 Mar 24;21(3):e0344023. doi: 10.1371/journal.pone.0344023 (PMC13012730; doi:10.1371/journal.pone.0344023)
Supplement: Attachment — Submitted filename: Author Response.docx [file pone.0344023.s001.docx]

Dear Editors and Reviewers:

Thank you for your letter and for the reviewers’ comments concerning our manuscript entitled “Traffic conflict identification method on curved road based on Frenet coordinate system” (ID: PONE-D-25-40226). Those comments are all valuable and very helpful for revising and improving our paper, as well as the important guiding significance to our research. We have studied comments carefully and have made correction which we hope meet with approval. The revised paragraphs are all highlighted in bold throughout the revised paper. Our response is outlined below, with the reviewer’s comments presented in italics for clarity. Responds to the reviewer’s comments:

*Reviewer #1: The topic is interesting and consistent with the scope of the journal. The authors propose some kind of "generalization" of surrogate measures of safety computation, switching from the Cartesian coordinate system to the Frenet. The latter is well-suited for both straight and curved road segments, whereas the former, as correctly pointed out by the authors, has several shortcomings when dealing with curved segments. The paper provides a fresh point-of-view to the computation of surrogate measures of safety, which is welcome; unfortunately, I believe that the paper could be improved with some additional tests.*

*1.Line 189, please better motivate the choice of 0.5 s.*

Thank you for your valuable suggestion. In the original manuscript, the selection of the 0.5s threshold was stated without a detailed explanation of the rationale. Actually, the determination of this threshold involves considerations of two aspects: an overly short time interval may lead to false positives due to trajectory drift, while an excessively long period could cause the segment to span two different states, thereby failing to capture the specific vehicle status. Accordingly, the 0.5s threshold was adopted based on its discriminative performance in practical applications. The justification for selecting this threshold has also been added to the revised manuscript.

It is worth noting that when the time interval is set too short, misjudgments may occur due to trajectory drift; when the time interval is set too long, the interval may span two states simultaneously, thereby losing the necessity of determining the vehicle's state. This paper adopts a 0.5-second time interval, which can largely avoid the two scenarios mentioned above in practical discrimination while accurately describing the vehicle's lateral and longitudinal motion on curved road sections.

*2. Lines 352-361. Please clarify if your centerline reference is the painted road centerline (i.e., you are assuming that the painted lines correspond to the actual midpoint axis of the road, which may be questionable). If so, there seems to be a non-negligible deviation from the spline and the painted centerline at certain points (Figure 13), how does this affect your results? Can you add some remarks on this?*

Thank you for your valuable comment.The road centerline reference was calibrated based on each lane marking (or road boundary line). Fig. 13 is a schematic diagram. Since the section shown is a transition zone within an expressway reconstruction and expansion work area, the road boundaries appear blurred with shadows, and some of the old markings have not been completely removed in practice. As a result, visual inspection may create the illusion of significant deviation between the fitted spline curve and the actual centerline of the markings at certain locations. This issue was considered during the calibration process of the road centerline and therefore does not affect the results. We have added clarifications regarding the centerline reference in the main text as follows:

When actually fitting the road centerline function, each lane line (or road boundary line) is considered collectively to calibrate the road centerline, thereby reducing errors introduced by the road centerline. Furthermore, considering lane direction variations and differences between lanes in the same direction during observation, multiple lanes in the same direction undergo calibration processing. After error compensation, the final calibrated road centerline is formed.

*3. Lines 397-402. The core of your work is to compare conflicts detected using the "traditional" and the "new" methods. Selecting the threshold to identify conflicts is a crucial operation that could significantly impact the results. Here, however, the TTC threshold is chosen in a rather arbitrary way. The authors could explore more of the literature to justify it, or to test several different thresholds.*

Thank you for your valuable suggestion. We have substantiated the rationale for threshold selection through literature citations and incorporated this into the main text.

Relatively speaking, the TTC severe conflict threshold of 3.0 seconds is more widely applied[27]. Moreover, validating with a larger threshold yields more representative overall results, as it avoids the limitation where the method only holds true under a specific small threshold. Therefore, this paper employs a 3.0-second threshold for rear-end and severe lane-change conflicts to identify severe conflicts, collecting only those with values below this threshold. Minor/general conflicts are excluded from consideration (hereafter, “conflict” and “severe conflict” refer exclusively to severe conflicts below 3.0 seconds).

*4. Figure 15, it could be better to split it into two graphs, one for the rear-end and one for the lane-change.*

Thank you for your valuable suggestion. We noted that integrating rear-end collisions and lane-change conflicts into a single graph better reflects the differences in conflict identification. Therefore, we chose to present the conflict count results in one graph. Upon the reviewer's reminder, we realized this approach also makes it difficult to intuitively discern the differences between following conflicts and lane-change conflicts from the graph. Consequently, in the main text, we split Fig. 16 into two sub-graphs, each displaying rear-end conflicts and lane-change conflicts respectively.

**(a) rear-end	(b) lane-change**

**Fig. 16 Comparison of the number of conflicts between the two methods**

*5. How to verify the superiority of the proposed method by comparing it to the traditional approach?*

Thank you for your valuable comment. In the results analysis, we demonstrated the superiority of the new method over the traditional method in four aspects.

- The new method identified more severe rear-end conflicts than the traditional approach in all non-straight segments, indicating a reduction in missed detections on curves. In contrast, the number of severe lane-changing conflicts remained largely consistent between the two methods. A paired-sample t-test confirmed that the difference in severe conflict counts—especially rear-end conflicts—was statistically significant.
- Further analysis using box plots of time- and segment-stratified data (8 groups × 10 segments) revealed that the largest increases in rear-end conflict detection occurred in segments 3, 4, 7, 8, and 9—transition zones between straight and curved sections. Minimal to no increase was observed in purely straight or purely curved segments.
- The traditional method misidentified some lane-changing conflicts outside the actual roadway in curved areas. The new method corrected this issue, successfully mapping 17 severe lane-changing conflict points back onto the road.
- Among the 125 additional severe rear-end conflicts identified by the new method, 10 involved dangerous vehicle states with deceleration rates exceeding -4 m/s² or -1.5 m/s², confirming that the method can detect previously overlooked risks.

*6. Some literature suggests updating to the latest literature.*

Thank you for your valuable comment. We have updated some of the references to include more recent literature from the past five years, ensuring the article's relevance to the current research frontier.

Reviewer #2:

*1. TTC is commonly used in the field of traffic safety to represent Time to Collision. Modify the Time to Conflict or its abbreviation in this article to avoid confusion. In addition, please unify the expression of “lane hanging” and “lane change” in the manuscript.*

Thank you for your valuable suggestion. In the revised draft, we have standardized the corresponding terminology throughout the text, including replacing “Time to Conflict” with “Time to Collision,” “lane changing” with “lane-change,” and replacing “lane switching” with “lane switch” during our comprehensive review.

*2. The current language standard in the manuscript falls below the journal's requirements. There are numerous grammatical errors and logical gaps between paragraphs. Professional editing is recommended. In addition, there are many confusing expressions that are difficult to understand, such as “It is necessary to wait until the accident Analysis can only be vehicleried out after the occurrence, which is ex post facto” in Line 39-40. Also, The numbering (e.g. (1) 1) ①) in this paper is confusing, please unify.*

Thank you for your valuable suggestion. We have made extensive revisions to the text, enhancing its logical flow and grammatical accuracy to meet the requirements of the target journal. Regarding your comment on lines 39-40, where the phrasing “requires waiting until after an accident occurs to conduct post-event analysis” was unclear, we have revised it to “the reactive nature of accident-based analysis leads to inherent delays in safety intervention” This better conveys the intended meaning. Additionally, following your recommendation, we have reorganized the numbering system throughout the text and standardized its formatting.

*3. The literature review lacks comprehensiveness, particularly regarding studies on traffic conflict identification at curved roads. Furthermore, more recent references should be included.*

Thank you for your valuable suggestion. We have supplemented the literature review with additional research on traffic conflicts at curves to enhance its comprehensiveness.

Tarko [17] proposed two alternative indicators, TD1 and TD2, for curves. However, most studies still implicitly transfer conflict detection assumptions from straight sections to curved sections without sufficient adjustment.

(1) The definition is unclear. In a curve, should “current direction” refer to the vehicle's instantaneous tangential direction (Direction 1) or the actual driving trajectory direction (Direction 2)? Existing research predominantly defaults to Direction 1 (i.e., tangential direction), while Direction 2 better aligns with the vehicle's actual driving path. Although Tarko [17] proposed TD2, which accounts for lateral motion, it has not gained mainstream adoption.

*4. Why choose the Frenet coordination system and what are its typical advantages compared to the Cartesian coordination system?*

Thank you for your valuable comment. In the introduction, we outlined two major issues in identifying traffic conflicts using curved segments: ambiguities in definitions and unreasonable definitions. These problems stem from using the Cartesian coordinate system to detect conflicts on curved segments. The Frenet coordinate system offers an alternative perspective on vehicle trajectories by treating curved paths as straight lines, thereby clarifying definitions and simplifying computational processes. In Section 1.1's introduction to the Frenet coordinate system, we briefly explained how the Cartesian coordinate system's definition of spatial points leads to inaccurate conflict identification. We acknowledge that the original phrasing may have been confusing, resulting in an inadequate explanation of this issue. Accordingly, we have revised the relevant text.

Traffic conflict identification relies on an appropriate coordinate system. To better identify traffic conflicts on curved road segments, it is essential to first establish a more suitable coordinate system. Typically, the Cartesian coordinate system is used to describe vehicle trajectory positions, as shown in Fig. 2a. However, on curved road sections, due to the influence of road curvature, defining potential collision points and travel directions in the Cartesian coordinate system may yield counterintuitive results, making it difficult to accurately describe vehicle trajectories. To address the limitations of the Cartesian coordinate system in conflict identification, this paper adopts a Frenet coordinate system for traffic conflict analysis. In the Frenet coordinate system, the "s"-axis aligns with the road centerline, representing the longitudinal travel distance of the vehicle along the road, while the "l"-axis, perpendicular to the s-axis, represents the lateral displacement distance. The Frenet coordinate system accounts for road curvature, and within this framework, vehicle trajectories are treated as straight lines, as illustrated in Fig. 2b. Compared to curved trajectories, straight trajectories are easier to analyze, thereby reducing the complexity of trajectory representation and conflict recognition. Additionally, this paper places reference points along the road centerline at 10-meter intervals and constructs a polynomial-fitted centerline through these points, as shown in Fig. 2c. A comparison between Fig. 2a and Fig. 2b clearly illustrates the differences between the two coordinate systems.

*5. It is suggested to supplement the overall architecture diagram of the proposed method in “1 Method”, presenting the technical framework and logical structure systematically to enhance the clarity of the discussion.*

Thank you for your valuable suggestion. Following your recommendation, we have added an overall architecture diagram to the “Methodology” section, systematically presenting the technical framework and logical structure to enhance the clarity of our discussion.

This section combines the Frenet coordinate system, vehicle status determination, and TTC indicators to introduce a complete method for implementing full-line (straight line + curve) collision detection using the Frenet coordinate system. The overall architecture diagram is shown below.

Fig. 2 Method architecture diagram

*6. Some symbols in the formula are not explained, which increases the difficulty of reading. In Figures 6-7, vehicle labeling and subscript capitalization are inconsistent.*

Thank you for your valuable suggestion. We have supplemented the explanations for certain symbols to ensure that all symbols in every formula are fully explained within the text. Additionally, Figures 6 and 7 have been updated to maintain consistent capitalization for vehicle labels and subscripts throughout the figures.

$l_{n-1}$：The length of the vehicle $n-1$，m；

$v_{\left( n-1 \right)x}$：The instantaneous vehicle speed x-axis component of the vehicle $n-1$ at time $t$，m/s；

**Fig. 7 Calculation of rear-end conflict of TTC indicator**

**Fig. 8 The traditional TTC indicator lane-change conflict calculation under the Cartesian coordinate system**

*7. In “2 Data”, the data processing part is too lengthy, it is recommended to simplify it. The result analysis part mainly compares the identification performance of the proposed method and the traditional method. How to obtain actual traffic conflicts based on the real data, and how accurate are the above methods compared to the actual conflicts?*

Thank you for your valuable suggestion. We have streamlined the data processing in Section 2 “Data,” simplifying numerous details with weaker relevance. The complete methodology for obtaining actual traffic conflicts from real data is detailed in Section 1 “Methodology.” Additionally, Section 2.4 provides a retrospective overview of the conflict identification processes for both methods. Both methods identify actual conflict data with high accuracy. Typically, in conflict studies, TTC identification serves as the actual conflict outcome, while other methods are used to detect potential omissions or errors, thereby assessing the accuracy of conflict identification. This paper uses the traditional method (TTC identification in the Cartesian coordinate system) as the baseline and demonstrates that the new method achieves improved accuracy in conflict identification results compared to the traditional approach.

*8. The contributions of this article are not clear both in the introduction and conclusion sections.*

Thank you for your valuable comment. We have revised the introduction and conclusion sections to better articulate the contributions of this paper.

*9. Some paragraphs are too long and not easy to read.*

Thank you for your valuable comment. Some overly long paragraphs have been condensed or split to ensure readability.

Thank you very much for your attention and time. Look forward to hearing from you.

Yours sincerely,

Jingan Wu

October 22, 2025
