# Supplementary material for: Traffic conflict identification method on curved road based on Frenet coordinate system
Source: PLoS One. 2026 Mar 24;21(3):e0344023. doi: 10.1371/journal.pone.0344023 (PMC13012730; doi:10.1371/journal.pone.0344023)
Supplement: Attachment — Submitted filename: Response to Reviewers208.docx [file pone.0344023.s002.docx]

Dear Editors and Reviewers:

Thank you for your letter and for the reviewers’ comments concerning our manuscript entitled “Traffic conflict identification method on curved road based on Frenet coordinate system” (ID: PONE-D-25-40226). Those comments are all valuable and very helpful for revising and improving our paper, as well as the important guiding significance to our research. We have studied comments carefully and have made correction which we hope meet with approval. the revised paragraphs are highlighted in red throughout the revised paper. Our response is outlined below, with the reviewer’s comments presented in bold for clarity. Responds to the reviewer’s comments:

*Reviewer #1: The authors have addressed all the concerns raised in the previous round, and the manuscript has been notably strengthened in terms of parameter justification, methodological validation, and literature support. A few minor suggestions are as follows:*

*1.* *The introduction and literature review could be further enriched by including key references that specifically outline research progress and existing methodological gaps in conflict identification on curved road sections.*

Thank you for your comment. While we devoted significant discussion in the Introduction and References to the limitations of conflict identification under the Cartesian coordinate system, the coverage of conflicts on curved road segments was indeed less thorough. Accordingly, we have restructured the entire Introduction. This revision involved adding a synthesis of recent advances and existing methodological gaps specifically related to conflicts on curved roads, while also condensing the original text to enhance its logical flow and readability.

In recent years, some scholars have addressed the issues of vehicle trajectory tracking and conflict identification on curved roads through various approaches. For example, Li described the motion state of vehicles on curves by combining the curve radius with the degree of steering angle deviation of the vehicle [22]. Hu integrated two different conflict indicators while accounting for their correlation, and applied a bivariate Bayesian hierarchical extreme value modeling method to comprehensively identify traffic risks on curves, thereby reducing the impact of individual indicators on risk assessment [23]. Cai utilized the relative angle between vehicles to accurately identify and warn of hazardous vehicles on both straight and curved sections [24]. A few scholars, such as Tarko [21], have considered two alternative indicators, TD1 and TD2, for curved road segments. However, in most traffic conflict studies, no distinct conflict identification method has been specifically proposed for curved sections; instead, conflict detection on curves is often equated with that on straight segments. Although the aforementioned methods for vehicle trajectory tracking and conflict identification on curves have shown effectiveness in specific scenarios or for particular problems, their underlying logic tends to provide tailored solutions for specific issues. As a result, they often struggle to adapt to varied scenarios and complex situations, and may involve computationally intensive procedures. The Frenet coordinate system, which is constructed based on the road centerline, simplifies the process of “fitting boundaries – tracking trajectories” for vehicle trajectory tracking and exhibits good adaptability to both straight and curved segments. Moreover, identifying traffic conflicts in the Frenet coordinate system allows for the continued use of classic traffic conflict indicators, thereby avoiding the issues of ambiguous and unreasonable conflict definitions mentioned earlier, and ensuring the accuracy and rationality of conflict identification. Additionally, given that traffic conflict techniques generally demonstrate good generalization across multiple scenarios, conflict identification within the Frenet framework also offers strong scenario adaptability. Furthermore, it can significantly reduce the computational complexity involved in vehicle trajectory and conflict analysis on curved sections [25]. [17]	Zhang, H., Gao, H., Ding, N., et al. 2025. Characterizing driver fingerprinting of new energy vehicles in risky scenarios: A naturalistic driving study. Green Energy and Intelligent Transportation, 100320.

[18]	Zhang, H., Luo, Y., Ding, N., et al. 2025. Evaluation of eco-driving performance of electric vehicles using driving behavior-enabled graph spectrums: A naturalistic driving study in China. Green Energy and Intelligent Transportation, 4(1), 100246.

[19]	Li, S., Zhang, H., Ding, N., et al. 2025. Associations of battery cell voltage consistency with driving behavior of real-world electric vehicles. Green Energy and Intelligent Transportation, 4(4), 100236.

[22]	Li, T., Ruan, J., Zhang, K. 2025. The investigation of reinforcement learning-based End-to-End decision-making algorithms for autonomous driving on the road with consecutive sharp turns. Green Energy and Intelligent Transportation, 100288.

[23]	Hu, Z., Wei, P., Sheng, L., et al. 2025. Bivariate Bayesian hierarchical extreme value modeling using multi-type traffic conflict for crash estimation on freeway horizontal curves. Accident Analysis & Prevention, 215, 108019.

[24]	Cai, X., Lv, B., Yao, H., et al. 2025. V-FCW: Vector-based forward collision warning algorithm for curved road conflicts using V2X networks. Accident Analysis & Prevention, 210, 107836.

[25]	Li, B., Zhang, Y M. 2020. Fast trajectory planning in Cartesian rather than Frenet frame: A precise solution for autonomous driving in complex urban scenarios. IFAC-PapersOnLine, 53(2):17306-17311.

*2.* *The theoretical contributions (e.g., proposing a unified framework for curved road conflict identification) and practical implications (e.g., supporting safety assessments in intelligent transportation systems) of this study should be summarized more explicitly.*

Thank you to the reviewers for pointing out the lack of clarity regarding the theoretical contributions and practical implications in the conclusion section. We have significantly strengthened this part in the revised version.

This study successfully addressed the critical limitations of traditional Cartesian coordinate-based traffic conflict measures in curved road segments, where ambiguous definitions and significant misidentification prevail. We proposed and validated a novel, integrated framework for conflict identification specifically designed for curved and transitional sections. The core theoretical contribution of this work is the establishment of a unified analytical framework that systematically combines the Frenet coordinate system with a precise vehicle state (lane-keeping/lane-changing) determination module and the Time-to-Collision (TTC) metric. This framework fundamentally resolves the definitional ambiguity and unreasonable motion assumptions inherent in applying traditional TTC to curves by decoupling vehicle motion into intuitive longitudinal (s) and lateral (l) components relative to the road geometry. It provides a consistent and mathematically rigorous foundation for describing vehicle interactions across both straight and curved alignments, moving beyond the domain-limited approaches of prior research.

The practical implications and actionable insights of this Frenet-based TTC framework are substantial for real-world road safety applications. Firstly, it enables accurate and consistent safety assessment across entire road networks, eliminating the previous methodological discontinuity between straight and curved segments. The framework's ability to correctly map conflict points onto the actual roadway and significantly reduce missed detections (particularly for rear-end conflicts in transition zones) provides transportation engineers and safety analysts with a more reliable tool for proactive hotspot identification, road safety audits, and before-and-after evaluation of geometric design or traffic control measures. Secondly, the output—high-fidelity, georeferenced conflict data—can directly inform the development and calibration of Advanced Driver Assistance Systems (ADAS) and vehicle-to-infrastructure (V2I) communication protocols for curved sections, enhancing their risk prediction and warning algorithms. The validation results, which showed the detection of previously overlooked dangerous events (e.g., conflicts involving high deceleration rates), confirm the framework's potential to uncover latent risks in existing infrastructure, thereby supporting more targeted and effective safety interventions.

In summary, the proposed framework offers a robust methodological advancement for traffic conflict analysis. While this study is limited by its focus on a specific site and time period, it establishes a validated proof of concept. Future work should expand validation to diverse road geometries, traffic conditions, and integrate with dynamic trajectory prediction models to address the inherent limitations of the Frenet system in high-curvature scenarios. This will further solidify its role as a practical tool for intelligent transportation systems aiming to enhance safety on roads of all configurations.

*3. The manuscript would benefit from additional language polishing.*

We sincerely appreciate the language refinement suggestions provided by the reviewers. We have meticulously proofread and optimized the entire manuscript, including adjusting sentence structures, standardizing academic terminology, and enhancing textual fluency. Additionally, we specifically invited native English-speaking research colleagues to assist in the review process to ensure clear, accurate, and professional language expression.

Reviewer #3:

*This paper proposes a traffic conflict detection method based on the Frenet coordinate system, aiming to address the identification bias of the conventional Time-to-Collision (TTC) metric on curved road segments caused by misalignment between the coordinate system and actual vehicle motion. The research topic is of clear practical relevance: with the increasing availability of high-precision trajectory data, accurately assessing microscopic traffic safety risks under complex road geometries has become a critical challenge in intelligent transportation systems and proactive safety applications. By adopting the Frenet coordinate system, the authors effectively decouple longitudinal and lateral vehicle dynamics on curves and demonstrate—using real-world trajectory data—the advantages of their approach in detecting severe conflict events such as rear-end collisions. Notably, the proposed method exhibits superior risk-capturing capability compared to traditional Cartesian-based methods, especially in transition zones between straight and curved segments.*

*The manuscript is generally well-structured and reflects a conscientious response to the reviewers’ previous comments. Terminology and language expression have been noticeably improved, particularly through substantial enhancements in the methodological framework diagram, symbol definitions, and consistency across figures and tables, all of which contribute to better readability.*

*Nevertheless, there remain several areas where the paper could be further strengthened. The specific modification suggestions are as follows:*

*1. Introduction needs to be further revised to highlight the existing research gaps.*

*The current introduction provides a general overview of traffic safety and conflict detection but fails to clearly articulate the specific limitations of existing Time-to-Collision (TTC) methodologies in curved road environments. While it mentions the reactive nature of crash-based analysis, it does not sufficiently foreground the core methodological gap—namely, that conventional TTC frameworks rooted in Cartesian coordinates are ill-suited for accurately capturing vehicle dynamics on curves due to misalignment between coordinate axes and actual travel direction.*

Thank you for your comment. The key limitations of conflict identification methods in curved road environments primarily fall into two aspects: first, the complexity and inaccuracy of trajectory tracking; second, the ambiguous and unreasonable definition of conflict indicators in curved road scenarios. In response, we have revised the Introduction to provide a clearer and more focused elaboration on these two issues.

Specifically, in the second paragraph of the Introduction, we have emphasized the complexities and deviations in trajectory tracking on curved segments. From the third to the fifth paragraphs, we systematically outline the categories of conflict indicators, definitional challenges, and issues arising during conflict identification, offering a detailed discussion to better highlight the deficiencies of existing research and the necessity for the proposed improvements.

In current traffic conflict research, the Cartesian coordinate system is predominantly adopted by default to determine the location of traffic conflict points. On straight road segments, straight-line equations can be used within the Cartesian framework to fit road boundaries, enabling relatively accurate and straightforward estimation and prediction of conflicts based on vehicle trajectory points. However, on curved road segments, due to the diversity of road curvature types, it is difficult to accurately fit road boundaries according to actual conditions. Moreover, when vehicle trajectories span both straight and curved sections, tracking trajectories using road boundaries becomes more complex [6]. The trajectory points of lane-changing vehicles are influenced by both the lane-changing maneuver and the curve equation. Particularly on curved sections where the lengths of inner and outer lanes differ and where the paths of the front and rear wheels deviate, accurately describing traffic conflicts becomes challenging [7]. Consequently, determining trajectory points for lane-changing vehicles on curves under the Cartesian coordinate system is complicated and unsuitable for precisely capturing the dynamic behavior of vehicles on curved segments, which undermines the reliability of conflict identification. Therefore, the accuracy of determining the trajectory point of the lane-change vehicle based on the curve in the Cartesian coordinate system cannot be guaranteed, and the applicability is poor. The Frenet coordinate system is based on the road centerline, which can greatly simplify the calculation of vehicle trajectories and conflicts under the curve road [8].

Additionally, traffic conflict detection techniques rely on conflict indicators for classification. Existing commonly used indicators primarily fall into three categories: ① Qualitative judgments based on evasive maneuvers (such as turning or significant deceleration) [9–11]; ② Measures based on spatio-temporal proximity [12–13]; ③ Indicators based on the motion characteristics of traffic entities, such as deceleration rate to avoid conflict (DRAC) [14–16]. Overall, the development of these traffic conflict indicators has significantly contributed to the promotion and application of traffic conflict techniques as well as to conflict identification. However, most existing indicators are primarily applied to ordinary straight road sections or urban intersections, while studies focusing on curved road segments remain scarce [17–19]. Currently widely used TTC and its derivative metrics (such as TIT, TET [20]), along with DRAC, are all based on a key assumption: “traffic participants maintain their current speed and direction unchanged.” Directly applying this assumption to curved sections (using highway transition zones as an example) raises the following issues:

*2. Why are minor accident cases not utilized? It is recommended to provide supplementary explanations.*

*The introduction points out that one of the limitations of the traditional accident-based data method is that "minor accidents or severe conflicts that do not lead to accidents are often not recorded, yet they contain a wealth of safety information." However, the main text only focuses on "severe conflicts" with TTC < 3.0s, completely excluding consideration of minor/general conflicts. It fails to illustrate whether the new method is capable of capturing such unrecorded events, nor does it explain how to use such information to supplement safety assessments, which inadequately connects with the original intention of "supplementing the traffic event model" mentioned in the introduction.*

Thank you for your comment. In conventional accident records, typically only incidents resulting in significant loss of life or property damage are documented, making it difficult to obtain complete data on minor accidents. According to the traffic event model, traffic events range from no conflict, minor conflict, moderate conflict, and severe conflict to traffic accidents—without a clear severity classification for accidents themselves. The model posits a strong correlation between severe traffic conflicts and traffic accidents, meaning that a severe conflict is likely to lead to an accident if no evasive action is taken.

Given the randomness and scarcity of accidents, the difficulty in recording all accident data, and the theoretical foundation of the traffic event model, it is common practice to use statistical parameters of **severe conflicts** to assess road traffic risk. In contrast, the association between minor/moderate conflicts and traffic accidents is weaker, as such conflicts often arise during normal car‑following or lane‑changing maneuvers. Therefore, relying on minor/moderate conflicts to gauge traffic risk would introduce inaccuracy.

For these reasons, this study adopts **severe conflicts** as the metric for evaluating conflict risk, thereby demonstrating the effectiveness of the Frenet coordinate system in identifying traffic conflicts.

*3. Additional explanations regarding the rationale for selecting the research road segment are required to strengthen the rationality of the sample.*

*The current study has not provided any elaboration on the rationale for the selection of the core research road segment. It only directly mentions that data was collected from a specific segment without explaining why this segment is suitable as a case for research on traffic conflicts on curved roads. Such an omission results in a lack of support for the scientificity and rationality of sample selection, leaving readers unable to judge whether the characteristics of this segment can represent the general attributes of the target research objects (e.g., curved roads), thereby affecting the credibility and promotional value of the research conclusions.*

Sincere thanks to the reviewer for the valuable feedback. The point you raised regarding the insufficient justification for the selection of the study road segment is indeed crucial, as it directly relates to the scientific validity of the sample and the generalizability of the conclusions. We fully agree with your observation and have made substantial additions to the manuscript accordingly. In Section 2.1, we have systematically elaborated on three core reasons for selecting the K283 section of the Jiqing Expressway as the research case: geometric representativeness, typicality and safety relevance of the operational context, and feasibility along with quality assurance of data collection. These aspects have now been clearly presented and addressed accordingly.

The data collection location is Jinan-Qingdao Highway, Shandong Province, China. This segment features a standard S-curve configuration as well as critical tangent-to-curve transition zones—precisely the core geometric scenarios where traditional methods fail and which this study aims to address. At the same time, the highway work zone, particularly the lane-switch area, provides a realistic and safety‑critical operational environment with specific constraints such as speed limits and lane‑changing maneuvers, making the conflict analysis more practically meaningful. The layout of the segment enables drone video to capture the entire vehicle trajectory with full, clear coverage, obtaining complete vehicle movement data for straight sections, curved sections, and straight‑to‑curve transition segments. Therefore, the curved road section studied in this paper takes the lane-changing construction zone of this expressway as an example.

*4. Literature review needs enhanced focus and integration of recent advances.*

*The literature review currently presents relevant studies in a somewhat fragmented manner and lacks critical synthesis, particularly regarding recent developments in trajectory-based conflict detection (post-2020). Although Tarko’s alternative indicators (TD1/TD2) are mentioned, the discussion does not adequately contextualize how these relate to—or differ from—the proposed Frenet-TTC framework.*

Thank you for your comment. We have supplemented the latest developments in trajectory-based conflict detection (post-2020) in the revised manuscript. By summarizing existing research on vehicle trajectory tracking and conflict identification on curved roads, we analyzed the strengths and limitations of their identification processes. While these methods demonstrate good performance in specific scenarios or for particular problems, their underlying logic—tailoring solutions to specific issues—makes them less adaptable to diverse scenarios and complex problems, often involving computationally intensive procedures. In contrast, the Frenet coordinate system offers advantages in simplifying trajectory tracking, maintaining compatibility with classical conflict indicators, and possessing strong scenario adaptability. Therefore, it provides more effective conflict identification.

In recent years, some scholars have addressed the issues of vehicle trajectory tracking and conflict identification on curved roads through various approaches. For example, Li described the motion state of vehicles on curves by combining the curve radius with the degree of steering angle deviation of the vehicle [22]. Hu integrated two different conflict indicators while accounting for their correlation, and applied a bivariate Bayesian hierarchical extreme value modeling method to comprehensively identify traffic risks on curves, thereby reducing the impact of individual indicators on risk assessment [23]. Cai utilized the relative angle between vehicles to accurately identify and warn of hazardous vehicles on both straight and curved sections [24]. A few scholars, such as Tarko [21], have considered two alternative indicators, TD1 and TD2, for curved road segments. However, in most traffic conflict studies, no distinct conflict identification method has been specifically proposed for curved sections; instead, conflict detection on curves is often equated with that on straight segments. Although the aforementioned methods for vehicle trajectory tracking and conflict identification on curves have shown effectiveness in specific scenarios or for particular problems, their underlying logic tends to provide tailored solutions for specific issues. As a result, they often struggle to adapt to varied scenarios and complex situations, and may involve computationally intensive procedures. The Frenet coordinate system, which is constructed based on the road centerline, simplifies the process of “fitting boundaries – tracking trajectories” for vehicle trajectory tracking and exhibits good adaptability to both straight and curved segments. Moreover, identifying traffic conflicts in the Frenet coordinate system allows for the continued use of classic traffic conflict indicators, thereby avoiding the issues of ambiguous and unreasonable conflict definitions mentioned earlier, and ensuring the accuracy and rationality of conflict identification. Additionally, given that traffic conflict techniques generally demonstrate good generalization across multiple scenarios, conflict identification within the Frenet framework also offers strong scenario adaptability. Furthermore, it can significantly reduce the computational complexity involved in vehicle trajectory and conflict analysis on curved sections [25].

[22]	Li, T., Ruan, J., Zhang, K. 2025. The investigation of reinforcement learning-based End-to-End decision-making algorithms for autonomous driving on the road with consecutive sharp turns. Green Energy and Intelligent Transportation, 100288.

[23]	Hu, Z., Wei, P., Sheng, L., et al. 2025. Bivariate Bayesian hierarchical extreme value modeling using multi-type traffic conflict for crash estimation on freeway horizontal curves. Accident Analysis & Prevention, 215, 108019.

[24]	Cai, X., Lv, B., Yao, H., et al. 2025. V-FCW: Vector-based forward collision warning algorithm for curved road conflicts using V2X networks. Accident Analysis & Prevention, 210, 107836.

[25]	Li, B., Zhang, Y M. 2020. Fast trajectory planning in Cartesian rather than Frenet frame: A precise solution for autonomous driving in complex urban scenarios. IFAC-PapersOnLine, 53(2):17306-17311.

*5.* *Conclusions need greater specificity on practical implications and limitations.*

*The conclusion section remains overly descriptive and does not sufficiently differentiate this study from prior work in terms of actionable insights. It should explicitly state how the Frenet-based TTC framework can inform real-world applications*

Thank you for pointing out that the conclusion section was overly descriptive and did not sufficiently highlight the actionable insights of our study compared to prior work. We have restructured the conclusion to better emphasize its practical significance.

In the revised version, we have dedicated a paragraph specifically to discussing "The Practical Implications and Actionable Insights of the Frenet-Based TTC Framework." We have detailed how this framework translates into actionable insights for traffic safety management practice—for instance, by providing engineers with a reliable tool for comprehensive road safety assessment to support decision-making, and by explaining how its high-fidelity, mappable conflict data serves as critical input for enhancing the performance of intelligent vehicles and vehicle-infrastructure cooperative systems in curved environments. These clarifications directly address the question of "how this research guides real-world application," delineating the pathway from methodological development to engineering practice, thereby more clearly demonstrating the applied differentiation and value of our work.

This study successfully addressed the critical limitations of traditional Cartesian coordinate-based traffic conflict measures in curved road segments, where ambiguous definitions and significant misidentification prevail. We proposed and validated a novel, integrated framework for conflict identification specifically designed for curved and transitional sections. The core theoretical contribution of this work is the establishment of a unified analytical framework that systematically combines the Frenet coordinate system with a precise vehicle state (lane-keeping/lane-changing) determination module and the Time-to-Collision (TTC) metric. This framework fundamentally resolves the definitional ambiguity and unreasonable motion assumptions inherent in applying traditional TTC to curves by decoupling vehicle motion into intuitive longitudinal (s) and lateral (l) components relative to the road geometry. It provides a consistent and mathematically rigorous foundation for describing vehicle interactions across both straight and curved alignments, moving beyond the domain-limited approaches of prior research.

The practical implications and actionable insights of this Frenet-based TTC framework are substantial for real-world road safety applications. Firstly, it enables accurate and consistent safety assessment across entire road networks, eliminating the previous methodological discontinuity between straight and curved segments. The framework's ability to correctly map conflict points onto the actual roadway and significantly reduce missed detections (particularly for rear-end conflicts in transition zones) provides transportation engineers and safety analysts with a more reliable tool for proactive hotspot identification, road safety audits, and before-and-after evaluation of geometric design or traffic control measures. Secondly, the output—high-fidelity, georeferenced conflict data—can directly inform the development and calibration of Advanced Driver Assistance Systems (ADAS) and vehicle-to-infrastructure (V2I) communication protocols for curved sections, enhancing their risk prediction and warning algorithms. The validation results, which showed the detection of previously overlooked dangerous events (e.g., conflicts involving high deceleration rates), confirm the framework's potential to uncover latent risks in existing infrastructure, thereby supporting more targeted and effective safety interventions.

In summary, the proposed framework offers a robust methodological advancement for traffic conflict analysis. While this study is limited by its focus on a specific site and time period, it establishes a validated proof of concept. Future work should expand validation to diverse road geometries, traffic conditions, and integrate with dynamic trajectory prediction models to address the inherent limitations of the Frenet system in high-curvature scenarios. This will further solidify its role as a practical tool for intelligent transportation systems aiming to enhance safety on roads of all configurations.

Thank you very much for your attention and time. Look forward to hearing from you.

Yours sincerely,

Jingan Wu

January 19, 2026
